# Pattern of medication utilization in hospitalized patients with COVID-19 in three District Headquarters Hospitals in the Punjab province of Pakistan

**DOI:** 10.1016/j.rcsop.2021.100101

**Published:** 2021-12-29

**Authors:** Zia Ul Mustafa, Chia Siang Kow, Muhammad Salman, Mahpara Kanwal, Muhammad Bilal Riaz, Samina Parveen, Syed Shahzad Hasan

**Affiliations:** a Department of Pharmacy Services, District Headquarter (DHQ) Hospital, Pakpattan 57400, Pakistan; b School of Pharmacy, Monash University Malaysia, Bandar Sunway, Selangor, Malaysia; c School of Postgraduate Studies, International Medical University, Kuala Lumpur, Malaysia; d Department of Pharmacy, The University of the Lahore, Lahore 54000, Pakistan; e Department of Pharmacy Services, District Headquarter (DHQ) Hospital, Okara South City, Pakistan; f Department of Pharmacy Services, District Headquarter (DHQ) Hospital, Chakwal, Pakistan; g Department of Pharmacy, School of Applied Sciences, University of Huddersfield, Huddersfield, United Kingdom; h School of Biomedical Sciences & Pharmacy, University of Newcastle, Callaghan, Australia

**Keywords:** COVID-19, SARS-CoV-2, Medication, Pakistan, Steroid, Heparin, Anticoagulant, COVID-19, Coronavirus Disease-2019, SARS-CoV-2, Severe Acute Respiratory Syndrome Coronavirus 2

## Abstract

**Purpose:**

In Pakistan, a wide range of repurposed drugs are recommended to manage hospitalized patients with COVID-19. Therefore, the current study was conducted to evaluate the pattern of utilization of repurposed drugs and other potential therapeutic options among hospitalized patients with COVID-19 in Pakistan.

**Methods:**

This retrospective, multicenter, descriptive study enrolled consecutive hospitalized patients with COVID-19 who were admitted between March 1, 2021, and April 30, 2021, from three District Headquarter Hospitals in the Punjab province of Pakistan. We described patient and clinical characteristics and medications, stratified by COVID-19 severity during hospitalization: mild, moderate, and severe. In addition, an analytical study of drug utilization was conducted.

**Findings:**

A total of 444 hospitalized patients with COVID-19 were included. Remdesvir, corticosteroids, antibiotics, and antithrombotics were administered to 45.0%, 93.9%, 84.9%, and 60.1% of patients, respectively. Specifically, dexamethasone was the most commonly used corticosteroid among the included patients (*n* = 405; 91.2%), irrespective of their clinical severity. Only 60.1% of patients hospitalized with COVID-19 in our cohort received antithrombotic therapy, and the prevalence of use was especially low (27.8%) in patients with mild illness. Of 444 patientsscreened, 399 (89.9%) patients had been discharged, and 45 patients (10.1%) died.

**Implications:**

We provided an important glimpse into the utilization patterns of several medications of interest for the treatment of COVID-19 in Pakistan, which had not been entirely evidence-based, especially concerning systemic corticosteroids and antibiotics.

## 1 Introduction

Since its emergence, nearly 1.2 million laboratory-confirmed cases of coronavirus disease-2019 (COVID-19) has been reported in Pakistan, resulting in more than 28,000 deaths.^1^ Currently, Pakistan is under the danger of the fifth COVID-19 wave (delta variant wave followed by emerging omicron variant) where significant number of cases are reported per day.

The substantial mortality with COVID-19 can be attributed to the emerging variants of concerns of the severe acute respiratory syndrome coronavirus 2 (SARS-CoV-2)^2^^,^^3^, a lack of effective treatment options, and non-preparedness to curb the pandemic. Most patients infected with SARS-CoV-2 experience mild to moderate illness requiring symptomatic treatment and home quarantine. However, older individuals with underlying chronic diseases are more likely to develop a severe course of the disease, necessitating hospitalization.^4^ There is thus far no conclusive effective treatment options available to prevent disease progression to severe illness. Symptom-based care and oxygen therapy if necessary, are provided to the hospitalized patients with COVID-19. Critical cases are being managed with invasive or non-invasive mechanical ventilation.^5^

Many drugs have been touted as potential treatment options for COVID-19. Repurposing or re-profiling, which is a process of identifying existing drugs for new indications, has been at the forefront of the approaches to deal with the COVID-19 pandemic. It is estimated that more than 75% of the existing drugs can be repurposed for novel indications.^6^ In Pakistan, a wide range of repurposed drugs - including dexamethasone, remdesivir, tocilizumab, and azithromycin are recommended for the management of hospitalized patients with COVID-19.^7^

Health care professionals in Pakistan are advised to manage hospitalized patients with COVID-19 according to the clinical guideline^7^ issued by the Ministry of National Health Services, Regulation and Coordination of Pakistan. Systemic corticosteroids which include dexamethasone, hydrocortisone, methylprednisolone, and prednisolone should only be administered to patients with severe COVID-19, while their use should be avoided in patients with mild-to-moderate illness.^7^ On the other hand, anticoagulants are advised in all hospitalized patients with COVID-19.^7^ As there is no role for antibiotics in the management of patients with COVID-19, their use should be restricted to those with confirmed secondary bacterial infections.^7^

Thus far, there has been no study which evaluated the pattern of the use of medications in hospitalized patients with COVID-19 in Pakistan. The current study was conducted to evaluate the pattern of utilization of repurposed drugs and other potential therapeutic options (e.g., azithromycin, remdesivir, ivermectin, enoxaparin, and corticosteroids) among hospitalized patients with COVID-19 in Pakistan.

## 2 Methods

### 2.1 Study design and population

This retrospective, multicenter, descriptive study enrolled patients admitted to the specialized COVID-19 wards in the three District Headquarters (DHQ) Hospitals in Pakpattan, Okara, and Chakwal respectively, of the Punjab Province, Pakistan, from March 1, 2021, to April 30, 2021. The population from the three districts are similar in demographic characteristics.

The inclusion criteria included patients aged 18 and above with laboratory-confirmed COVID-19. A positive laboratory finding for COVID-19 was defined as a positive result on a real-time reverse-transcriptase polymerase chain reaction assay of nasal or pharyngeal swab specimens. Patients with negative or indeterminant results (unclear PCR results) were excluded since they were managed differently than patients with laboratory-confirmed COVID-19. In addition, pregnant women were also excluded.

The clinical severity of included patients with COVID-19 was defined using the clinical practice guideline for the management of COVID-19 issued by the Ministry of National Health Services, Regulation, and Coordination, Government of Pakistan.^7^ Mild cases were those with symptoms consistent with COVID-19 but without hemodynamic instability or requirement for oxygen therapy, and with normal chest x-ray findings. Those with mild infiltrates (<50% involvement of lung fields) observed on chest X-ray and with oxygen saturation ≥ 94% were classified as moderate cases. Severe cases were those with oxygen saturation < 94% on room air at the sea level, respiratory rate of > 30 breaths/min, arterial oxygen partial pressure (PaO_2_)/ fractional inspired oxygen (FiO_2_) < 300 mmHg, or lung infiltrates > 50% observed on chest X-ray.

This study was conducted by following the Declaration of Helsinki (revised in 2013). The study protocol was approved by the Ethics Committee of the School of Pharmacy, the University of Lahore, Pakistan (REC/DPP/FOP/37). We also obtained approval from the DHQ Hospitals (No. 5739/PA/DHQ) to retrieve their medical records.

### 2.2 Drug utilization and clinical endpoints

All data for this study were retrospectively extracted from the medical records of DHQ hospitals, which contained information on demographics (age, sex), disease severity, medications used, and clinical progress notes. Upon extraction, a clinical pharmacist reviewed all data for accuracy and completeness.

We assessed medications given to the included patients during their hospitalization, including (1) medications repurposed for the treatment of COVID-19, defined as pharmacological agents under investigation or reported to have potential effects against COVID-19, such as remdesivir, azithromycin, and ivermectin; (2) medications that may be used for supportive care in patients with COVID-19, including corticosteroids, antimicrobials/antibiotics, and anticoagulants; and (3) miscellaneous medications that may be of interest to patients with COVID-19, including statins, cetirizine, montelukast, and omeprazole.

We selected these medications for investigation a priori according to previously cited evidence and hypotheses. We included all routes of administration (unless otherwise specified), although these medications are most commonly administered intravenously or orally. We collected the medication use data as a dichotomous variable, where we recorded whether the patient ever received the medication (even only once) during their inpatient encounter(s) for COVID-19. The ascertainment of drug utilization started on the index date and continued until the occurrence of clinical endpoints of interest, namely inpatient death or non-fatal discharge from the hospital. We also collected the two demographic variables available on the medical records, namely age and sex.

### 2.3 Statistical analysis

We described patient and clinical characteristics and the use of medications, stratified by COVID-19 severity during hospitalization and clinical endpoints (discharged/death). Frequency and percentage were reported for categorical variables. On the other hand, median with interquartile range (IQR) and mean with standard deviation were reported for continuous variables. A Chi-squared test was used to determine statistical differences between clinical endpoints and medication utilization. A two-sided *P* value of less than 0.05 was considered statistically significant. The statistical analyses were performed using SPSS version 25.

## 3 Results

A total of 444 laboratory-confirmed patients with COVID-19 admitted to the three DHQ hospitals in the Punjab Province were included in this study: 131 patients from DHQ hospital in Pakpattan, 184 patients from DHQ hospital in Okara, and 129 patients from DHQ hospital in Chakwal. Table 1 depicts the patient and clinical characteristics of the included patients, stratified by highest clinical severity during hospitalization. Among the 444 patients with COVID-19, 169 (38.1%) patients were with mild illness, 113 patients (25.5%) were with moderate illness, and 162 patients (36.5%) were with severe illness. The included patients had a median age of 56.0 (49.0–62.0), and most were females (*n* = 262; 59.0%). The majority of patients recovered and were discharged from the hospital (*n* = 399; 89.9%), while 45 patients (10.1%) died during hospitalization.

**Table 1:** Patient and clinical characteristics stratified by clinical severity.

**Item**	**Total (*n*** **=** **444)** **(*n*, *%*)**	**Mild (*n*** **=** **169)** **(*n, %*)**	**Moderate (*n*** **=** **113)** **(*n, %*)**	**Severe (*n*** **=** **162)** **(*n, %*)**
**Hospital**				
DHQ Pakpattan	131 (29.5)	12 (7.1)	28 (24.8)	91 (56.2)
DHQ Okara South	184 (41.4)	148 (87.6)	32 (28.3)	4 (2.5)
DHQ Chakwal	129 (29.1)	9 (5.3)	53 (46.9)	67 (41.4)

**Sex**				
Male	182 (41.0)	41 (24.3)	60 (53.1)	81 (50.0)
Female	262 (59.0)	128 (75.7)	53 (46.9)	81 (50.0)

**Age group**				
Median (IQR)	56.0 (49.0–62.0)	57.0 (49.5–60.0)	56.0 (50.0–62.5)	55.5 (46.0–66.3)
18–39	44 (10.0)	13 (7.7)	10 (8.8)	21 (13.0)
40–59	216 (48.6)	91 (53.8)	59 (52.3)	66 (40.7)
60 or more	184 (41.4)	65 (38.5)	44 (38.9)	75 (46.3)

**Inpatient mortality**				
No	399 (89.9)	169 (100)	111 (98.2)	119 (73.5)
Yes	45 (10.1)	0 (0)	2 (1.8)	43 (26.5)

DHQ = District Headquarters Hospital; IQR = interquartile range.

### 3.1 Utilization of repurposed drugs for the treatment of COVID-19

Overall, remdesivir (*n* = 200; 45.0%) was the most common repurposed drug received by the hospitalized patients with COVID-19, followed by azithromycin (*n* = 128; 28.8%) and ivermectin (*n* = 73; 16.4%). Remdesivir was most frequently received by the patients with moderate illness (*n* = 62; 54.9%) and severe illness (*n* = 104; 64.2), and the daily dose used ranged from 100 mg to 300 mg for a duration of up to seven days. Over half of the included patients (*n* = 91; 53.8%) with mild illness received azithromycin, and the daily dose ranged from 250 mg to 500 mg for five days. One-third of the patients (*n* = 54) with severe illness received ivermectin, and the daily dose ranged from 12 mg to 36 mg for up to seven days. Only one patient (0.6%) with severe illness received tocilizumab during hospitalization (see Table 2).

**Table 2:** Utilization of medications stratified by clinical severity.

Drug	Overall (n = 444)	Mild (n = 169)	Daily dose in mg unless otherwise specified (days)	Moderate (n = 113)	Daily dose in mg unless otherwise specified (days)	Severe (n = 162)	Daily dose in mg unless otherwise specified (days)
	**Repurposed drugs**						
Ivermectin	73 (16.4)	5 (3.0)	12–36 (2–6)	14 (12.4)	10–36 (1–5)	54 (33.3)	12–36 (1–6)
Remdesivir	200 (45.0)	34 (20.1)	100–200 (1–7)	62 (54.9)	100–300 (1–6)	104 (64.2)	100–300 (1–7)
Azithromycin	128 (28.8)	91 (53.8)	250–500 (5)	13 (11.5)	250–500 (5)	24 (14.8)	500 (5)
Tocilizumab	1 (0.2)	0	–	0	–	1 (0.6)	400 (1)

	**Corticosteroids**						
Dexamethasone	405 (91.2)	156 (92.3)	4–24 (1−10)	99 (87.6)	4–32 (1−12)	150 (92.6)	4–24 (1–15)
Hydrocortisone	17 (3.8)	3 (1.8)	250–750 (5)	4 (3.5)	100–500 (1–5)	10 (6.2)	250–500 (1–5)
Inhaled budesonide (puffs - 400 μg per puff)	135 (30.4)	9 (5.3)	4-6p (2–7)	53 (46.9)	4-6p (1–10)	73 (45.1)	4-6p (1–15)
Methylprednisolone	8 (1.8)	1 (0.6)	80 (4)	2 (1.8)	80 (1–2)	5 (3.1)	40–80 (1–6)
**At least one corticosteroid**	417 (93.9)	158 (93.5)	–	105 (92.9)	–	154 (95.1)	–
**Repurposed drugs + corticosteroids**	283 (63.7)	103 (60.9)	–	64 (56.6)	–	116 (71.6)	–

	**Antibiotics**						
Ceftriaxone (dose in g)	168 (37.8)	101 (59.8)	2 (1–8)	33 (29.2)	2 (1–10)	34 (21.0)	2 (1–10)
Moxifloxacin	202 (45.5)	35 (20.7)	400 (1–8)	68 (60.2)	400–500 (1−11)	99 (61.1)	400–800 (1–12)
Piperacillin-tazobactam (dose in g)	66 (14.9)	9 (5.3)	1.35–4.5 (1–5)	19 (16.8)	0.675–4.5 (5–8)	38 (23.5)	0.675–4.5 (1–9)
Meropenem (dose in g)	50 (11.3)	6 (3.6)	2 (5–7)	13 (11.5)	2–3 (3−12)	31 (19.1)	1–3 (1−13)
**At least one antibiotic**^a^	377 (84.9)	132 (78.1)	–	98 (86.7)	–	147 (90.7)	–
**At least one antibiotic**^b^	399 (89.9)	152 (89.9)	–	98 (86.7)		149 (92.0)	

	**Antithrombotic**						
Heparin	2 (0.5)	0	–	1 (0.9)	–	1 (0.6)	–
Enoxaparin	26 (5.9)	1 (0.6)	40 (5)	7 (6.2)	60 (1)	18 (11.1)	40–120 (1–10)
Rivaroxaban	184 (41.4)	5 (3.0)	10–30 (1–14)	65 (57.5)	15–30 (1–14)	114 (70.4)	10–30 (1–15)
Aspirin	66 (14.9)	33 (19.5)	75	25 (22.1)	75	8 (4.9)	75
**At least one antithrombotic**	267 (60.1)	47 (27.8)	–	91 (80.5)	–	129 (79.6)	–

	**Miscellaneous**						
Statin	9 (2.0)	0	–	5 (4.4)	–	4 (2.5)	–
Cetirizine	10 (2.3)	3 (1.8)	–	3 (2.7)	–	4 (2.5)	–
Montelukast	209 (47.1)	13 (7.7)	–	71 (62.8)	–	125 (77.2)	–
Omeprazole	347 (78.2)	141 (83.4)	–	93 (82.3)	–	113 (69.8)	–

a Without azithromycin.

b Inclusive of azithromycin.

### 3.2 Utilization of corticosteroids

Corticosteroids had a very high usage rate (overall: *n* = 417; 93.9%) among hospitalized patients with COVID-19 across the whole spectrum of clinical severity; 93.5% (*n* = 103) of patients with mild illness, 92.9% (*n* = 105) of patients with moderate illness, and 95.1% (*n* = 154) of patients with severe illness received at least one corticosteroid during hospitalization for COVID-19. Specifically, dexamethasone was the most commonly used corticosteroid among the included patients (*n* = 405; 91.2%), irrespective of their clinical severity. Dexamethasone was used in 92.3% (*n* = 156) of patients with mild illness, 90.7% (*n* = 147) of patients with moderate illness, and 92.6% (*n* = 150) of patients with severe illness, at a daily dose ranging from 4 mg to 32 mg for a period of up to 15 days. On the other hand, inhaled budesonide was mainly received by patients with moderate illness (*n* = 53; 46.9%) and patients with severe illness (*n* = 73; 45.1%), at a daily dose of 4–6 puffs for a maximum duration of 15 days. A total of 283 patients (63.7%) received a combination of the repurposed drug(s) and corticosteroid(s).

### 3.3 Utilization of antibiotic

A total of 377 patients (84.9%) hospitalized with COVID-19 received least one antibiotic. Utilization of antibiotics was found in 78.1% (*n* = 132) of patients with mild illness, 86.7% (*n* = 98) of patients with moderate illness, and 95.1% (*n* = 154) of patients with severe illness. Even higher proportion of patients with mild illness (*n* = 152; 89.9%) and patients with severe illness (*n* = 149; 92.0%) received at least one antibiotic if azithromycin was included in the analyses. Apart from azithromycin, the most frequently utilized antibiotic was moxifloxacin, which was received by 45.5% of the included patients. Other antibiotics utilized were ceftriaxone (*n* = 168; 37.8%), piperacillin-tazobactam (*n* = 66; 14.9%), and meropenem (*n* = 50; 11.3%).

### 3.4 Utilization of antithrombotic therapy

Over half of the patients (*n* = 267; 60.1%) hospitalized with COVID-19 received antithrombotic therapy. Antithrombotic therapy was mainly utilized in patients with moderate illness (*n* = 91; 80.5%) and in patients with severe illness (*n* = 129; 79.6%). The most frequently utilized antithrombotic was rivaroxaban (*n* = 194; 43.7%), with a daily dose ranging from 10 mg to 30 mg for a duration of up to 15 days. The majority of the patients (*n* = 170; 87.6%) received rivaroxaban at a dose (more than 10 mg daily) higher than that indicated for venous thromboembolism prophylaxis. Other anticoagulant prescribed were heparin (*n* = 2; 0.5%) and enoxaparin (*n* = 26; 5.9%). The daily dose of enoxaparin ranged from 40 mg to 120 mg; 10 out of 26 patients (38.5%) received enoxaparin at a dose (more than 40 mg daily) higher than that indicated for the prophylaxis of venous thromboembolism. On the other hand, aspirin was used in 66 patients hospitalized with COVID-19 (14.9%).

### 3.5 Utilization of miscellaneous medications that may be of interest

Over three-quarter of patients (*n* = 347; 78.2%) hospitalized with COVID-19 received omeprazole, predominantly in patients with mild illness (*n* = 141; 83.4%) and in patients with severe illness (*n* = 93; 82.3%). On the other hand, about half of the included patients (*n* = 209; 47.1%) hospitalized with COVID-19 utilized montelukast, primarily in patients with moderate illness (*n* = 71; 62.8%) and in patients with severe illness (*n* = 125; 77.2%).

### 3.6 Utilization of medications stratified by clinical endpoints

Table 3 presents the utilization of medications among patients who survived to discharge and among patients who died during hospitalization. A higher proportion of patient who survived to discharge received the repurposed drugs: ivermectin (17.5% versus 6.7%, *P* = 0.062), remdesivir (46.1% versus 35.6%; *P* = 0.177) and azithromycin (31.3% versus 6.7%; *P* = 0.001), compared to those who died during hospitalization. There was no significant difference in the prevalence of at least one corticosteroid use between those who survived to discharge and those who died during hospitalization (93.5% versus 95.6%; *p* = 0.999). However, a significantly higher proportion of those who survived to discharge received a combination of the repurposed drug(s) and corticosteroid(s) (67.4% versus 40.0%; *p* = 0.005). In patients who survived to discharge, the prevalence of use of at least one antimicrobial (84.0% versus 93.3%; *P* = 0.123) was higher, and the prevalence of use of at least one antithrombotic was lower (56.9% versus 88.9%; *p* = 0.050), compared to patients who died during hospitalization, though both did not reach statistical significance.

**Table 3:** Utilization of medications stratified by clinical endpoints.

**Drugs**	**Discharged (n** **=** **399)**	**Deceased (*n*** **=** **45)**	**Chi-square** ***p*-value**
**Repurposed drugs**			
Ivermectin	70 (17.5)	3 (6.7)	0.062
Remdesivir	184 (46.1)	16 (35.6)	0.177
Azithromycin	125 (31.3)	3 (6.7)	0.001
**Corticosteroids**			
Dexamethasone	363 (91.0)	42 (93.3)	0.583
Hydrocortisone	10 (2.5)	7 (15.6)	0.001
Budesonide Inhaler	100 (25.1)	35 (77.8)	0.001
Methylprednisolone	4 (1.0)	4 (8.9)	0.001
**At least one corticosteroid**	373 (93.5)	43 (95.6)	0.999
**Repurposed drugs + corticosteroids**	269 (67.4)	18 (40.0)	0.005
**Antimicrobials**			
Ceftriaxone	161 (40.4)	7 (15.6)	0.001
Moxifloxacin	167 (41.9)	36 (80.0)	0.001
Piperacillin-Tazobactam	63 (15.8)	3 (6.7)	0.103
Meropenem	45 (11.3)	5 (11.1)	0.973
**At least one antimicrobial**	335 (84.0)	42 (93.3)	0.123
**Antithrombotic**			
Enoxaparin	18 (4.5)	8 (17.8)	0.003
Rivaroxaban	160 (40.1)	34 (75.6)	0.001
Aspirin	62 (15.5)	4 (8.9)	0.235
**At least one antithrombotic**	227 (56.9)	40 (88.9)	0.050
**Miscellaneous**			
Statin	8 (2.0)	1 (2.2)	0.923
Cetirizine	10 (2.5)	5 (11.1)	0.012
Montelukast	175 (43.9)	35 (77.8)	0.001
Omeprazole	310 (77.7)	38 (84.4)	0.280

## 4 Discussion

To the best of the authors' knowledge, this is the first study investigating the utilization of medications in patients with COVID-19 admitted to Pakistani hospitals. Since the outbreak of COVID-19, several drugs have been investigated in patients with COVID-19, with few demonstrated positive outcomes. Therefore, we summarized in Table 4 the medications with positive outcomes across the randomized controlled trials conducted among patients with COVID-19.

**Table 4:** Drugs with positive results in COVID-19.

Drug	Recommended dosing regimen	Suitable at what stage	Randomized trial with positive outcome	Positive outcome	Potential risk	Evidence
Dexamethasone	Severe: 6 mg orally or IV infusion once daily for up to 10 days (or until hospital discharge if sooner) Critical: 20 mg IV once a day for 5 days, then 10 mg IV once a day for 5 days	Severe-to-critical	RECOVERY^8^	Mortality	Hyperglycaemia, delayed viral clearance	Effective
			CoDEX^32^	Clinical recovery		
Ivermectin	0.4 mg/kg (maximum 24 mg) once daily for 4 days	Mild-to-severe	IRCT20200408046987N1^15^	Mortality	–	Inconclusive
			NCT04668469^16^			
Remdesivir	IV 200 mg loading dose on day 1, followed by a 100 mg maintenance dose administered daily on days 2 through 10 or until hospital discharge	Moderate-to-severe	ACTT-1^18^	Time to clinical recovery	AKI, transaminitis	Possibly effective
				Mortality		
Budesonide	DPI 800 μg twice daily for 14 days	Mild	PRINCIPLE^11^	Time to clinical recovery	–	Possibly effective
			STOIC^12^			
Methylprednisolone	IV 250 mg once daily 3 days	Severe	IRCT20200404046947N1^33^	Mortality	Hyperglycaemia, delayed viral clearance	Possibly effective
Heparin	Enoxaparin: SC 1 mg/kg twice daily Unfractionated heparin: IV infusion to target for aPTT of 1.5 to 2.5 times the upper limit of normal	Mild-to-critical	ATTACC, ACTIV-4a, and REMAP-CAP multi-platform trials^26^^,^^27^	Thromboembolic events	Beeding	Possibly effective

We observed a high rate of corticosteroid usage in our cohort of hospitalized patients with COVID-19, which should prompt attention to the Pakistani clinicians. The clinical efficacy of dexamethasone in patients with COVID-19 has been established only in those with severe illness (Table 4); a large, randomized open-label trial in the United Kingdom (RECOVERY trial^8^) with oral or intravenous dexamethasone demonstrated reduced 28-day mortality among hospitalized patients with COVID-19 receiving invasive mechanical ventilation or extracorporeal membrane oxygenation at baseline (relative risk: 0.64; 95% confidence interval 0.51–0.81) as well as hospitalized patients on noninvasive oxygen therapy (relative risk: 0.82; 95% confidence interval 0.72–0.94), but no significant benefits among patients who did not require either oxygen or ventilatory support (relative risk: 1.19; 95% confidence interval 0.91–1.55).

Therefore, patients with mild to moderate illnesses should not be prescribed dexamethasone and other systemic corticosteroids. Even in patients with severe illness, a dose of 6 mg daily is adequate based on the RECOVERY trial,^8^ but we observe a prescription of up to four times higher than the recommended daily dose. Clinicians should be reminded that the use of systemic corticosteroids is not without harms, where it can be associated with hyperglycemia and an increased risk of secondary infections (including bacterial, fungal, and Strongyloides infections), where potential mortality benefits could be negated.^9^^,^^10^ In patients with mild-to-moderate illness, perhaps the use of inhaled budesonide can be encouraged based on the findings from the PRINCIPLE trial^11^ and the STOIC trial^12^ (Table 4), which demonstrated significantly faster recovery than to usual care among outpatients with COVID-19. Nevertheless, 1600 μg per day of inhaled budesonide is recommended (used in the PRINCIPLE trial and STOIC trial), but patients in our cohort received up to 2400 μg per day.

Ivermectin had been repurposed for the treatment of COVID-19 after it demonstrated in vitro inhibitory action against SARS-CoV-2.^13^ Currently, ivermectin has been used as a part of the treatment strategies in hospitalized patients with COVID-19 in various countries, including Pakistan.^7^ However, we demand that ivermectin be used with caution in patients with COVID-19. While the use of ivermectin was associated with mortality benefits in hospitalized patients with COVID-19 as reported in a recent systematic review and meta-analysis^14^; thus far, the significant reduction in the risk of mortality is demonstrated in only two trials^15^^,^^16^ (Table 4) which are available as preprints (not peer-reviewed). Pending more evaluation of its clinical efficacy, large-scale, adequately powered, bias-minimized, randomized controlled trials of ivermectin should be encouraged, especially in low-and-middle-income countries, including Pakistan, which might not be able to afford more costly antiviral treatment such as remdesivir or monoclonal antibodies.

Remdesivir is a novel nucleotide analog that demonstrates in vitro antiviral activity against SARS-CoV-2.^17^ Remdesivir should be recommended for hospitalized patients with severe COVID-19 (receiving low-flow oxygen supplementation) because the ACTT-1 trial^18^ (Table 4) reported that it reduced time to recovery (median of seven versus nine days; relative risk for recovery: 1.45; 95% confidence interval 1.18–1.79) and risk of death (hazard ratio: 0.30; 95% confidence interval 0.14–0.64) compared to placebo. Therefore, the use of remdesivir is appropriately justified in our cohort of patients with severe illness. Furthermore, we opined that reduced time to recovery should be regarded as an important clinical endpoint since it could conserve valuable health care resources, especially when there is a widespread COVID-19 outbreak.

However, the use of remdesivir is probably not necessary in those with mild-to-moderate illness. In the ACTT-1 trial,^18^ remdesivir did not appear to reduce the time to recovery among the 119 patients with mild-to-moderate illness (i.e., no hypoxia or tachypnea; median five versus six days; relative risk for recovery: 1.29; 95% confidence interval 0.91–1.83). In addition, in the WHO-sponsored SOLIDARITY trial^19^ among patients hospitalized with COVID-19, there was no significant difference in the overall 28-day mortality between patients randomized to remdesivir and patients randomized to standard care (relative risk: 0.95; 95% confidence interval 0.81–1.11). About one-fifth of patients with mild illness and over half of patients with moderate illness in our cohort received remdesivir.

Antibiotics should not be recommended to treat any viral infection, including COVID-19, and secondary bacterial infection does not appear to accompany the course of COVID-19 predominantly. However, since it might be difficult to distinguish the clinical features of COVID-19 from bacterial pneumonia, empirical coverage for bacterial pneumonia is reasonable, especially if the diagnosis is ambiguous. Empirical coverage for bacterial pneumonia may also be reasonable if there is high clinical suspicion (e.g., new consolidation on chest imaging in patients with COVID-19). Importantly, as recommended by the Dutch Working Party on Antibiotic Policy,^20^ sputum and blood cultures should be obtained prior to initiating empirical antibiotic therapy in patients with COVID-19 who have radiological findings and/or inflammatory markers compatible with bacterial co-infection to confirm the diagnosis. They also emphasize the need for appropriate de-escalation of antibiotic therapy based on the results of culture testing.

The inappropriate use of antibiotics is reported worldwide among patients with COVID-19 without secondary bacterial infection. A recent systematic review and meta-analysis^21^ investigating the use of antibiotics among patients with COVID-19 reported that almost three-quarters (74.6%; 95% confidence interval 68.3–80.0%) of patients with COVID-19 received antibiotics, although the prevalence of bacterial co-infection in this patient population was merely 8.6% (95% confidence interval 4.7–15.2%). A previous Pakistani study^22^ reported that the use of many classes of antibiotics had been increased during the COVID-19 pandemic compared to the corresponding pre-pandemic period, especially azithromycin, which demonstrated the highest increment in its utilization. We found a high rate of azithromycin usage in our cohort of patients with mild illness, which is a cause of concern because acquired macrolide resistance has been an increasingly recognized problem even before the COVID-19 pandemic. The use of azithromycin should be discouraged; a systematic review and meta-analysis^23^ of randomized controlled trials reported no improvement in 28-day all-cause mortality (odds ratio: 0.96; 95% confidence interval 0.88–1.05) and the need for invasive mechanical ventilation in patients (odds ratio: 0.96; 95% confidence interval 0.49–1.87). The high usage rate of moxifloxacin (a fluoroquinolone) and ceftriaxone (a third-generation cephalosporin) should also be a concern since it strongly correlates with the risk for infection by Methicillin-resistant *Staphylococcus aureus*^24^ and infection by extended-spectrum-β-lactamase (ESBL)-producing organisms,^25^ respectively.

The many microvascular and macrovascular thrombotic complications evident in hospitalized patients with COVID-19, particularly among severely ill subpopulations, indicate the need to prescribe antithrombotic/anticoagulants to reduce the risk of development of thromboembolic events. In fact, the current recommendation favors the use of pharmacologic prophylaxis of venous thromboembolism for all hospitalized patients with COVID-19.^7^ However, only 60.1% of patients hospitalized with COVID-19 in our cohort received antithrombotic therapy, and the prevalence of use was especially low (27.8%) in patients with mild illness. The use of anticoagulants is important, but the intensity of anticoagulation is also of special relevance. Randomized controlled trials^26^^,^^27^, ^28^ have demonstrated the therapeutic value of higher-intensity anticoagulation with heparin for the prophylaxis of venous thromboembolism in mild-to-moderate^27^ and severe^26^ patients, respectively, where the rate of major thrombotic events was reduced with therapeutic dosing (1.4% versus 2.7% for mild-to-moderately ill 5.3% versus 10.7% for severely ill patients) compared with prophylactic dosing, and without significantly increased risk of major bleeding. Nevertheless, only 38.5% of patients in our cohort who received enoxaparin were prescribed higher-intensity dosing (more than 40 mg daily).

While the rate of usage of higher-intensity dosing of rivaroxaban (more than 10 mg daily) is higher (87.6%), therapeutic dosing rivaroxaban may not be a justifiable option, since in the recently reported ACTION trial^29^, the risk of the composite outcome of thromboembolic events was not significantly different with therapeutic-dosing rivaroxaban versus the prophylactic-dosing rivaroxaban (relative risk: 0.75; 95% confidence interval 0·45–1·26), but with a significantly higher risk of major or clinically relevant non-major bleeding (relative risk: 3.64; 95% confidence interval 1·61–8·27). Heparins, such as enoxaparin, unlike rivaroxaban, have anti-inflammatory effects and could therefore mitigate the thrombo-inflammation in patients with COVID-19.^30^ On the other hand, randomized controlled trials^26^^,^^27^ administered anticoagulants for hospitalized patients with COVID-19 up to 14 days or until recovery (hospital discharge or liberation from supplemental oxygen for ≥24 h), and as such this duration should have been followed unless clinically significant bleeding develops. In our cohort of patients receiving anticoagulants, the duration of anticoagulation used ranges from merely one day to as long as 15 days.

We observed a high rate of usage of omeprazole, a proton-pump inhibitor, in our cohort of patients hospitalized with COVID-19. However, there have been suggestions of a worse prognosis from observational studies^31^ using proton-pump inhibitors in patients with COVID-19, which should discourage their widespread use pending data from clinical trials. Indeed, the use of proton-pump inhibitors for the prophylaxis of stress ulcers should be reserved for critically ill patients (receiving intensive care) who are assessed as high risk for gastrointestinal bleeding.

There are some limitations to be noted. Firstly, we included only three DHQ hospitals within the Punjab Province of Pakistan, and thus the findings may not be generalized to the whole country. Secondly, we could not record other patient and clinical characteristics since there was no proper documentation in the available medical records. However, the available data permitted assessment of medication utilization based on clinical severity.

## 5 Conclusion

This is the first descriptive study in Pakistan to explore the medication use pattern in hospitalized patients with COVID-19. We provided an important glimpse into the utilization patterns of several medications of interest for the treatment of COVID-19, which had not been entirely evidence-based, especially concerning systemic corticosteroids and antibiotics. Although there is a knowledge gap in many of the medications used to treat COVID-19, the evidence-based pharmacotherapy for patients with COVID-19 is still too important to be foregone to optimize valuable health care resources during the pandemic.

## Declaration of Competing Interest

The authors declare that they have no known competing financial interests or personal relationships that could have appeared to influence the work reported in this paper.
